# Editorial: Vestibular Contributions to Health and Disease

**DOI:** 10.3389/fneur.2018.00117

**Published:** 2018-03-19

**Authors:** Bernard Cohen, Richard Lewis

**Affiliations:** ^1^Department of Neurology, Mount Sinai School of Medicine, New York, NY, United States; ^2^Department of Otolaryngology, Harvard Medical School, Boston, MA, United States; ^3^Department of Neurology, Harvard Medical School, Boston, MA, United States

**Keywords:** vestibular, physiology, pathophysiology, labyrinth, disease

**Editorial on the Research Topic**

**Vestibular Contributions to Health and Disease**

## Introduction

The inter-related functions of several systems that evolved hundreds of millions of years ago, the vestibular, sympathetic, and cardiovascular systems, have important clinical implications in modern neurology and neuro-otology. Operating largely out of consciousness, the vestibular system responds to head and body movement in any direction, stabilizes the head and body with regard to gravity in three-dimensional space, and maintains vital bodily functions through an interaction of vestibular-related components of the brainstem and cerebellum with the cardiovascular and sympathetic systems.

We conceived this volume as an opportunity to highlight some of the basic science and clinical findings that have arisen over the first years of the twenty-first century with regard to these systems. Our concept was to have several broad topics: 1. fundamental vestibular, brainstem, and cerebellar science; 2. new approaches to vestibular diagnosis, pathology, and treatment; and 3. investigations of the vestibular impact on the cardiovascular and sympathetic systems. Topics include the function of the otolith organs, evaluation of the vestibulo-ocular reflex (VOR) in pathologic conditions, and the vestibular input to sympathetic functions such as the vestibulo-sympathetic reflex (VSR). Motion sickness, a vestibulo-sympathetic interaction, is considered both historically and in terms of its typical clinical presentation. Also included are summaries of a little known receptor system, the skin sympathetic nerve activity (SSNA), which forms an important part of the sympathetic “fight or flight” response, and a new hypothesis about the neural basis for the Mal de Debarquement syndrome (MdDS).

## Vestibular Physiology—Basic Science

Ward et al. demonstrate that large *Tesla* magnets used in MRI scanners activate the peripheral end organ in a manner that replicates a constant angular acceleration. Thus, the MRI magnet allows one to study adaptation to this type of stimulus and simultaneously provides a way to identify, modify, or remove the vestibular activation that contaminates “resting” functional MRI scans.

Curthoys et al. demonstrate that otolith activity can be functionally segregated into static or sustained responses and transient responses, with the latter transduced primarily by type I hair cells. They show how this static-dynamic dichotomy could be clinically relevant, with particular reference to vestibular responses measured in the extra-ocular muscles and evoked with skull vibration (ocular vestibular-evoked myogenic potential).

McCall et al. provide an extensive review of the inputs and outputs of the vestibular system, particularly directed toward vestibulospinal and reticulospinal contributions. A large literature demonstrates significant differences in the functional characteristics of vestibulospinal and reticulospinal projections in alert and anesthetized cats and non-human primates and emphasizes the complexity of the terminations in neural groups adjacent to the motor neurons.

Yakushin et al. (1) found that vestibular only (VO) neurons in the vestibular nuclei are responsible for the shift of the axis of eye rotation toward gravity. The VO neurons did not modulate with head position and most likely underlie the direct pathway linking the vestibular end organ with the ocular motor nuclei. When tested in three dimensions, the VO neurons on either side of the brainstem responded differently to ipsilateral and contralateral rotations and were insensitive to drowsiness, in contrast to oculomotor-related vestibular neurons.

## Vestibular Pathophysiology—Understanding, Diagnosing, and Treating Vestibular Disorders

*The pathophysiology* of vestibular compensation is considered in a paper by Batuecas-Caletrio et al. They studied outcomes in patients undergoing surgical resection of vestibular nerve schwannomas and observed that the characteristics of re-fixation saccades correlated with compensation.

Frejo et al. address the pathophysiology of Meniere’s disease, classifying patients with bilateral disease based on co-morbidities such as migraine or autoimmune disease. They propose that this form of phenotyping may help segregate the different mechanisms that result in the Meniere’s syndrome and could eventually be linked to specific genetic syndromic diagnoses.

van den Burg et al. demonstrated an exciting new development in the diagnosis of Meniere’s disease, namely imaging that identified anatomic abnormalities in this illness. Specifically, enlargement of the semicircular canals was observed, a finding that mirrors the earlier results of Hallpike 80 years ago (2).

Dumas et al. show that vibration can elicit evidence of vestibular asymmetry in patients with peripheral deficits, even when they are fully compensated. The underlying physiology is not certain, but the authors argue that both canal and otolith afferents are activated by skull vibration. This technique could offer a quick new way to estimate the presence of semicircular canal lesions without rotational apparatus or caloric stimulation.

Halmagyi et al. review their technique for characterizing canal function and analyzing lesions of individual semicircular canals using the video head impulse test. Rapid head movement and observation of the evoked eye movements are much closer to the natural function of the VOR. As a result, head impulse testing using video goggles to measure eye movement responses has become relatively omnipresent in vestibular clinical laboratories. This paper reviews the experience to date with this method including its shortcomings and potential new uses.

The history of the superior canal dehiscence syndrome (SCD), which is now approaching two decades after its initial description by Lloyd Minor, is reviewed by Ward et al. This paper summarizes the clinical presentation, diagnosis, and therapeutic options for SCD. Because it can be treated surgically by inactivating the abnormal canal or closing the defect in its roof, it is essential that this diagnosis be considered when the clinical signs suggest its presence.

Karmali et al. examine how self-motion perceptual thresholds relate to age and fall risk, and find that while all of the tested angular, linear, and tilt thresholds that were measured increase with age, only roll tilt thresholds have a meaningful correlation with fall risk.

Dai et al. review the results of treatment of the MdDS in 141 patients. Their discovery of the first successful treatment for the MdDS, reported in 2014, has led to extensive experience treating this disorder which they report here. They found that the treatment was initially successful in 75% of patients, although efforts still must be made to stabilize the patients better in the time after treatment (Dai et al.).

The neural mechanism underlying the MdDS is considered by Cohen et al. They posit that MdDS had arises after prolonged exposure to roll tilts causes a lateral shift of the pitch orientation vector in the cerebellar nodulus. This activates VO neurons in the vestibular nuclei and causes them to oscillate at 0.2 Hz, resulting in the continuous sensation of rocking, swaying, and/or bobbing, which are characteristic of MdDS.

## Vestibulo-Sympathetic Interactions

A major theme of vestibular research in the twenty-first century has been expanding the realm of studies to include the frequently overlooked vestibular effects on the sympathetic nervous system, which helps control blood pressure, heart rate, sweating, and other basic behaviors. There are *two distinctly different components* to the VSR. *One component*, using glutamatergic transmission, involves the otolith system and the vertical semicircular canals [Holstein et al.; (3); Yakushin et al.; (4)]. Vestibular stimulation, i.e., upward, linear acceleration of the head and body produced by standing causes constriction of peripheral blood vessels in the legs by activating muscle sympathetic nerve activity (MSNA) (5–8).

Studying the effect of sinusoidal galvanic vestibular stimulation (sGVS) on anesthetized rats, Cohen and colleagues found that such stimulation initially caused a drop in BP and HR, a vasovagal response that is the precursor of neurogenic syncope (9). Interestingly, the vasovagal response disappeared with continued bouts of stimulation with sGVS, which was associated with a change in the response of BP and HR. The authors raise the possibility that such vestibular stimulation potentially could be used to thwart the occurrence of neurogenic syncope.

*A second VSR system* originates largely in the lateral and vertical semicircular canals and the otolith organs and projects into other aspects of the sympathetic system, using serotonin as the transmitter to initiate anxiety, sweating, and fear (10–14). Hammam and Macefield studied SSNA, a little known set of receptors activated by vestibular input that are critical for generating phenomena associated with motion sickness. Activated by weak linear acceleration along the horizontal plane, the SSNA is responsible for pallor and sweating. In comparison with the MSNA, which activates BP upon standing, the SSNA is also activated by linear acceleration, but the strength of the activation is orders of magnitude weaker than that elicited by MSNA. A striking finding was that SSNA is associated with the symptoms of motion sickness, namely nausea and vomiting, as well as with skin pallor and sweating.

Motion sickness, produced by activation of this system, has been an accompanying feature of travel for centuries. Huppert et al. provide a comprehensive review of motion sickness in their historical context. It is clear that the ancient Chinese and Greeks were well aware that body motion was responsible for “cart” sickness and seasickness, but the actual provocative motions were still a mystery. The authors review the incidents of both Chinese and European history when such seasickness proved to be a vital force that helped decide the course of battles and the fates of various nations.

Recent experiments have come closer to understanding the nature of the provocative motions responsible for producing nausea and motion sickness. The causes of motion sickness on tilting trains (15) were revisited, with refined apparatus by Bertolini et al. who argue that specific motion patterns, namely those that elicit Coriolis forces (which are generated when one moves within a moving reference frame), are particularly likely to elicit symptoms of motion sickness. An earlier study on tilting trains (15) concluded that motion sickness in the passengers came when the roll tilt of the cars was initiated after the train had entered into the curve. This implicated roll as the exciting source of the motion sickness, confirming the findings of Griffin and colleagues (16, 17). Bertolini et al. were able to show that motion sickness was dependent on starting the roll after the rotation had begun, as in the earlier study (15), showing that it was a combination of linear acceleration and roll that had caused the motion sickness.

## Conclusion

This collection of papers provides insight into the current state of vestibular research at levels ranging from basic physiology to clinical treatment. In particular, we feel that vestibular-autonomic interactions, which have been relatively neglected by much of the vestibular community, will ultimately prove to be an area of much significance for both the normal and pathologic influences of the vestibular system on behavior.

## Author Contributions

BC and RL contributed equally to this manuscript.

## Conflict of Interest Statement

The authors declare that the research was conducted in the absence of any commercial or financial relationships that could be construed as a potential conflict of interest.
